# Exploring the Effect of Virtual Education on Student Pharmacists’ Confidence toward APPE Readiness

**DOI:** 10.3390/pharmacy10050118

**Published:** 2022-09-22

**Authors:** Edith Mirzaian, Samara Jasmine White, Mona Karim, Kari L. Franson, Maryann Wu, Ying Wang

**Affiliations:** Titus Family Department of Clinical Pharmacy, University of Southern California School of Pharmacy, Los Angeles, CA 90033, USA

**Keywords:** APPE readiness, virtual education, experiential education, students’ confidence

## Abstract

A drop in confidence in Advanced Pharmacy Practice Experience (APPE) readiness was observed in students in the Class of 2022 prior to starting APPEs. We aim to investigate potential causes of students’ low confidence in APPE preparedness to provide solutions and to prevent this outcome with future students. We evaluated students’ perceived confidence to start APPEs and compared this to curricular changes, employment obligations, and the impact of COVID-19 on delivery of the pre-APPE and APPE curriculum. Students’ low confidence with APPE readiness was not indicative of the following factors: (1) delivery of the didactic curriculum, (2) students’ performance in the didactic curriculum, or (3) number of summative assessments in key didactic courses. Rather, the low confidence perception may have been due to differences such as a fully remote didactic experience in the P3 year, more virtual Introductory Pharmacy Practice Experiences (IPPEs), a reduced course load in the P3 spring semester, and changes to a pre-APPE preparatory course compared to other class years. The students’ self-reported midpoint scores during their first APPE block and preceptor’s evaluations on their performance contrasted their pre-APPE perceptions. Frequent in-person and on-site skills assessments throughout the didactic curriculum seem to reinforce confidence before APPEs.

## 1. Introduction

Modern pharmacy profession education in the United States (US) includes both didactic and experiential components. The experiential component consists of Introductory Pharmacy Practice Experiences (IPPEs) and Advanced Pharmacy Practice Experiences (APPEs) in which the provision of patient care is the central focus. The 2016 Accreditation Council for Pharmacy Education (ACPE) Standards requires that the pre-APPE curriculum provides the necessary foundation for student pharmacists to be prepared for APPEs [1]. While IPPE students are typically entrusted to perform tasks with direct and proactive supervision by their preceptors, APPE students are entrusted to perform activities that are more complex with indirect and reactive supervision [2,3].

Researchers who studied APPE readiness have found that beyond the requisite of knowledge and skills-based abilities, students’ APPE readiness also consisted of learner characteristics, workplace participation, and relationship-building skills [4]. Learner characteristics included a student’s personal qualities, such as self-awareness, adaptability, and professionalism, in addition to their clinical foundational knowledge, skills and experiences. Workplace participation was noted to be important for a student’s ability to quickly integrate themselves to the new APPE workplace environment, facilitated by the alignment and adoption of core entrustable professional activities (EPAs) in the IPPE and APPE curricula by specifying students’ roles and responsibilities at the start of APPEs [4,5]. Relationship-building skills were also reported as a key factor for interprofessional practice readiness during APPEs [4]. All of these components are expected to be a part of the pre-APPE curriculum, but are also supplemented by co-curricular activities and external employment opportunities. It is interesting to note that when other researchers introduced a new curricular track with the hope of improved APPE readiness, they found an increase in student confidence without a measured change in knowledge or preceptor perception. However, the increase in confidence was associated with the students positively rating their learning experience in the new curricular track [6].

Since the mandate of the Doctor of Pharmacy degree, there has been no bigger change in pharmacy curricula than the changes that resulted from the COVID-19 pandemic. It has been previously reported in the literature that the shift to remote education and healthcare has had a profound impact on pharmacy students’ learning [7]. One study found that their students reported either no or a negative effect on their learning as a result of the shifts in education [8]. The pandemic also impacted pharmacy student mental health which resulted in increased stress and difficulties with concentration [9]. With such a dramatic change, it is not a surprise that many pharmacy educators are still trying to assess the full scope of the impact of the pandemic on pharmacy education. Some authors go so far as to call for “innovation and rethinking the paradigm of how pharmacy programs educate and prepare students for pharmacy practice” [10].

**Figure 1:**
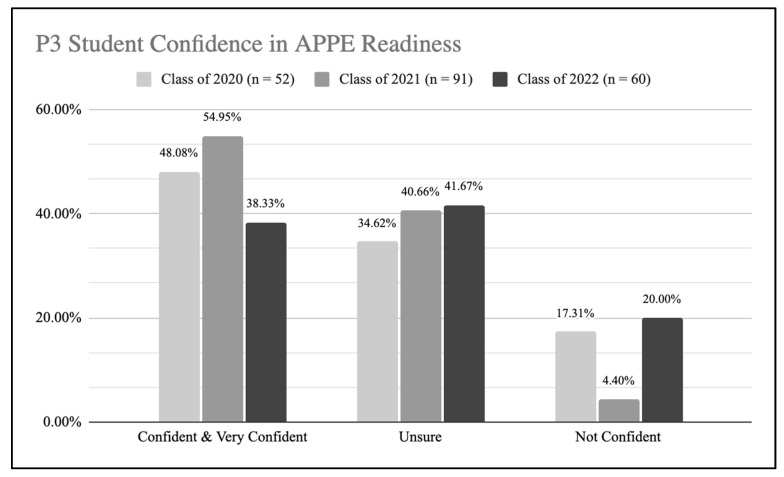
P3 student confidence with starting APPEs.

It was with this lens that the pharmacy educators at the University of Southern California (USC) School of Pharmacy viewed our APPE readiness data from the third year (P3) class in 2022 versus the previous two years. For the Class of 2020 and 2021, the P3s had been increasingly confident to enter their APPEs. Then in 2021, 16.6% fewer of the P3 students stated they were confident to enter their APPEs and there was a 15.6% increase in P3 students stating they were not confident (Figure 1). The primary purpose of this research is to assess the impact of the pandemic on the pre-APPE curriculum and factors that may have led to a decrease in students’ self-reported confidence on APPE readiness, with the secondary outcome to identify how to intervene and improve student self-reported confidence on APPE readiness.

## 2. Materials and Methods

Upon identifying this decrease in confidence for APPE readiness, the authors decided to compare the educational experiences between students in the Class of 2022 and students in the preceding two cohorts. The obvious difference was completing the full P3 academic year virtually; however, our objective was to identify other factors throughout the pre-APPE experience that may have contributed to their low confidence. The APPE Readiness Survey served as a source of information for not only student confidence in starting APPEs, but also confidence in achieving educational program outcomes, specific pharmacy topics and content areas, and the number of hours and settings students worked during the academic year. Academic and curricular factors were reviewed and compared between the Classes of 2020, 2021 and 2022.

A descriptive quantitative analysis was conducted on the data collected from the survey and other potential factors to evaluate whether the differences in experiences led to a decrease in student reported APPE readiness for the Class of 2022 compared to the Class of 2020 and 2021.

This study was approved and determined exempt by the Institutional Review Board (IRB) of the University of Southern California. 

### 2.1. APPE Readiness Survey

The USC School of Pharmacy APPE Readiness Survey was developed in 2012 in Qualtrics™ (Qualtrics LLC, 333 W. River Park Drive, Provo, UT 84604, USA), a web-based survey platform/tool, and is distributed to all P3 students via email every year in the Spring at the end of their didactic curriculum. The survey includes nineteen questions and assesses several areas including basic demographics, work settings, hours per week worked during P1–P3 years, plans upon graduation, confidence with knowledge of ACPE Standards Appendix 1 topics, with ability-based outcomes (ABOs), and overall confidence with APPE preparation [1]. The survey addresses students’ experiences with various methods of instruction and engaged learning in the curriculum and also provides an opportunity for students to provide open text feedback on their perceived preparedness level for APPEs.

Once distributed, the APPE Readiness Survey was active for up to fourteen days. Students who completed the survey were entered into a raffle to win prizes as an incentive. Survey responses were collected and analyzed by the Assistant Dean of Assessment then shared at assessment and curriculum committee meetings.

### 2.2. Academic Considerations

The following factors were reviewed and compared between the Classes of 2020, 2021 and 2022: curricular content and content placement, teaching and learning methods, academic workload, format of summative assessments, IPPEs, and differences in academic performance. Curricular content and placement was reviewed by examining four-year academic course plans and course structure and academic workload difference noted. Automated Approach to Reviewing and Developing Valuable Assessment Resources for your Curriculum (AARDVARC^©^, USC School of Pharmacy, Los Angeles, CA 90033, USA), our syllabi management system, was used to access and review course syllabi across the years [11]. Four-year academic course plans for each cohort were used to identify course structure and academic workload differences. Academic records were used to identify aggregate grade point averages and course performance scores for each cohort during the P3 year.

## 3. Results

### 3.1. Survey Results

The APPE Readiness Survey was completed by 52 students from the Class of 2020 (*n* = 186), 91 students from the Class of 2021 (*n* = 182), and 60 students from the Class of 2022 (*n* = 199), which resulted in a response rate of 27.96%, 50.00%, and 30.15% for each cohort, respectively. In the assessment of overall APPE readiness confidence levels, there was a major decrease for the Class of 2022 compared to the Classes of 2020 and 2021. The Class of 2022 reported lower percentages in ‘confident and very confident’ responses (38.33%), compared to the Classes of 2020 and 2021, 48.08% and 54.95%, respectively (Figure 1). A higher percentage of students in the 2022 cohort reported ‘not confident’ (20%) compared to previous Classes of 2020 and 2021, 17.31% and 4.4%, respectively.

**Figure 2:**
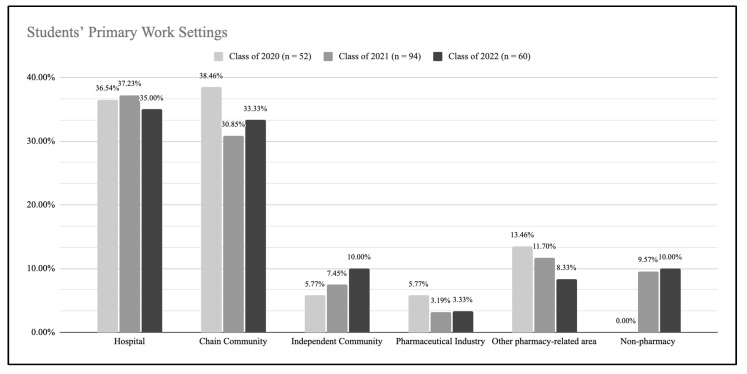
Students’ primary work settings from P1 through P3 years.

When observing the work settings and hours of students, the survey results indicated that students in all three cohorts had an even distribution of primary settings of work (Figure 2). Chain community and hospital pharmacy were the most represented employment settings among the students in the three classes (2020, 2021 and 2022). Students in non-pharmacy work settings represented roughly 10% of students in the classes of 2022 and 2021, and 0% from class of 2020. 

**Figure 3:**
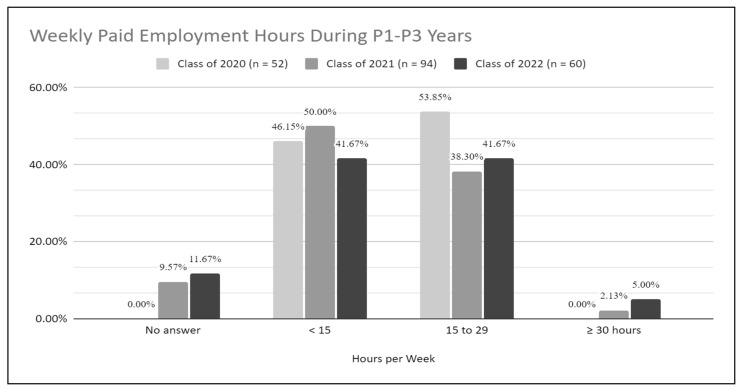
Students’ weekly paid employment hours during P1–P3 years.

The class of 2022 had the highest percentage of students working ≥30 h per week (5.00%) compared to the class of 2021 (2.13%) and the class of 2020 (0.00%), but overall they reported working fewer hours per week (Figure 3).

Despite the reported low confidence with starting APPEs, the students’ level of confidence for each ability-based outcome (ABO) was high for most ABOs (Table 1). Students expressed less confidence or were unsure of their confidence in certain areas such as financial and human resources, innovation and entrepreneurship, solving therapeutic problems and collaborating with interprofessional teams.

Regarding the students’ confidence in their knowledge of ACPE Appendix 1 topics [1], fewer students from the Class of 2022 reported feeling ‘confident’ or ‘very confident’ compared to the Class of 2021 with a difference of more than 10% in the following areas: human physiology, pharmaceutical calculations, healthcare systems, human anatomy, and patient assessment (Table 2).

When asked about their confidence in the P1–P3 didactic curriculum, students in the Class of 2022 reported more than 74% confidence in assessing patients and providing evidence-based best practice, and more than 83% in communication and acting in a trustworthy manner (Table 3). These results were still lower than those reported for students from the Classes of 2021 and 2020.

### 3.2. Curricular Changes

The Class of 2022 experienced differences in their P1–P3 didactic curriculum compared to the Classes of 2020 and 2021. The major difference was that the Class of 2022 completed the entirety of their P3 year remotely with virtual instruction due to the COVID-19 pandemic. Some P3 courses for the Class of 2022 were different than those of the previous cohorts due to intentional curricular changes. For instance, the Class of 2022 experienced a health sciences interprofessional education event virtually instead of in person, an APPE Gateway course that was more focused on the application of skills for APPE success, a reduced course load in the P3 Spring semester, and increased frequency of low-stakes assessments in courses to ensure engagement with the content and more open-book summative assessments designed to assess critical thinking and decision-making. In addition, the virtual instruction course schedule was vastly different than the schedule the students were accustomed to prior to remote learning [12]. The curricula experienced between the three groups were not similar by courses, structure and delivery. 

### 3.3. Academic Performance

The Class of 2022 demonstrated good overall academic performance at the end of their didactic curriculum with an average grade point average (GPA) of 3.60 at the end of the P3 year. Therapeutics of Cardiovascular Disorders and Therapeutics of Infectious Diseases were two major courses in the P3 curriculum. These courses cover topics frequently encountered by students on APPEs, particularly in the core required APPEs. In reviewing the course syllabi for the two courses, we observed that they included comparable types of summative assessments, contact time and assignments for the Classes of 2021 and 2022. Class of 2022 had more homework and pre-class quizzes compared to 2020 and 2021 in Therapeutics of Infectious Diseases and Therapeutics of Cardiovascular Disorders had one additional exam during the course. The cumulative GPA at the conclusion of their didactic curriculum was 3.43 for the Class of 2021 and 3.74 for the Class of 2022. The APPE Gateway is our school’s best mechanism to assess APPE readiness in our P3 students. The course has a multi-station OSCE which each student must successfully complete. All students from all cohorts were able to pass this course and successfully move on to the APPE year. 

### 3.4. APPE Evaluations and Performance

**Figure 4:**
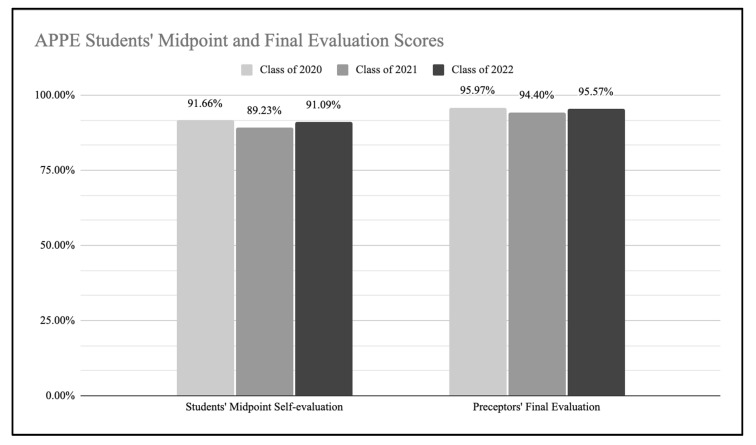
Student self-assessment midpoint and preceptor final evaluation of APPE students.

Students from the Class of 2022 rated their own performance at a high level when completing their midpoint self-evaluation for their first APPE block, with an average score of 91.09% (Figure 4). Their preceptors also rated their performance at a high level in the final evaluation, with an average score of 95.57%. Both averages were consistent with previous years. 

When taking a closer look into the final evaluations completed by preceptors for all non-community APPEs, there was again a disconnect from the low confidence levels reported by the students in the survey. The average scores students received for each category of the evaluation, which are directly aligned with the school’s ability-based outcomes (ABOs), were rated consistently between the two highest scores of 4 and 5, which translated to most students being rated “above average” or “excellent” for their performance in each criterion of the evaluation (Table 4).

The Class of 2022 students also performed well on their community APPEs, which include a performance evaluation different than the non-community APPEs and based on EPAs [5]. The average score on the preceptor final evaluations was 97.03%, well above the 80% required to receive credit for the rotation. The average score for the global assessment category was 2.91, which shows that the majority of students exceeded expectations by receiving a score of 3 for the APPE. Only a few students received a minimal passing score of 2, and 1 student from the Class of 2022 received a score of 0 (not passing) and was required to repeat the rotation.

## 4. Discussion

Twenty percent of students in the Class of 2022 reported they were not confident in their APPE readiness. However, the students’ pre-APPE perception of their readiness was not aligned with their self-reported midpoint performance during their first APPE block, nor the preceptors’ feedback on their performance. Changes in curricular structure and delivery, employment and challenges in experiential education due to the pandemic may have led to this reduced confidence. However, the Class of 2022 experienced many positive changes in the overall curriculum including self-paced learning, more engaged class sessions with application-based activities, more frequent low-stakes assessments throughout the year to ensure learning and retention of content, a reduced academic workload in the Spring semester, and an APPE Gateway course more focused on skills for APPE success compared to previous cohorts who experienced more focus on didactic review of major disease states encountered in inpatient and community-based patient care settings. 

Prior to remote instruction in the 2020–2021 academic year, the P1–P3 didactic schedule included four days per week of in-person instruction with both morning and afternoon sessions on each of those days, and one day dedicated to IPPEs. In contrast, during virtual instruction, each didactic course was allocated one 3 h synchronous session block either weekly or every other week. The synchronous sessions for each curriculum year were scheduled over two days of the week, with some fewer credit hour courses scheduled on alternating weeks. The two remaining days of the week were dedicated to assessments, virtual office hours with faculty, question and answer sessions, time for students to complete asynchronous work, and one day per week for IPPEs. All knowledge level instructional content was delivered in the asynchronous format with the synchronous sessions dedicated to application-based learning, engaging students with the content expert faculty applying and reinforcing the content from the asynchronous material [12]. It was our belief based on previous research that active learning would increase self-confidence [13,14].

The P3 Spring curriculum includes the APPE Gateway course which is designed to strengthen and reinforce skills required for APPE readiness. For previous classes, the APPE Gateway course included high-yield topic review with an opportunity to apply the knowledge and skills during an objective structured clinical examination (OSCE) at the end of the course. In Spring 2021, the APPE Gateway course focused primarily on the application of skills related to high-yield topics that are critical to APPE success. During the course, students created and delivered an in-service presentation, exercised presenting patients to preceptors, documented simulated patient encounters in various practice settings, exercised communication and difficult conversations with clinician peers as well as patients and caregivers, and applied evidence-based guidelines and protocols to patient scenarios. As a result of this structural change, the perception of the students in the Class of 2022 was that they didn’t receive a thorough “review of topics” in APPE Gateway like their peers even though all critical skill exercises were built around high-yield clinical topics. One option in this course may be to use reflective portfolios to increase student confidence as reported by Er et al. [15].

Another difference that the Class of 2022 experienced was a reduced course load in the P3 Spring curriculum, which was already a shortened semester (ten weeks) compared to a traditional semester (15 weeks). The Classes of 2020 and 2021 had six core courses in the P3 Spring curriculum, two of which were 5-unit therapeutics courses. In a curricular adjustment process, the content of one of those therapeutics courses was moved to other courses in the P2 curriculum. Therefore, the change resulted in a minimum of five core courses with a single Therapeutics course (Oncology and Immune Disorders). The academic workload in Spring of the P3 year for the Class of 2022 was reduced as a result of this change.

The asynchronous/synchronous instruction format allowed for increased frequency of engaging students with course material by implementing low-stakes assessments throughout each course including pre-, during and post-session quizzes, polls, and reflections [12]. The number of summative assessments, midterm and final examinations remained consistent compared to previous years; however, all were administered remotely. Examinations and quizzes were administered via secure exam software and with time limitations and students completed their exams at home. In most courses, summative examinations were written as open-book exams reinforcing critical thinking and decision-making, whereas traditionally, the exams for those courses were closed-book secure exams administered in a proctored classroom. It has been shown that while open versus closed book exams can lead to similar results [16,17,18,19], it has also been found that anticipating open-book exams leads to reduced study time and long-term retention [19]. Other assessments such as OSCEs were also conducted remotely but with virtual facilitation and evaluation by faculty, whereas traditionally, the OSCEs would occur in person in one-on-one interactions without access to notes.

The pandemic also impacted a major IPE event for the Class of 2022 during which students of various health professions work together in small groups to examine a patient case and develop a care plan for the patient as a team. IPE simulation activities are known to increase student confidence with IPE competencies [20]. Previous classes were able to participate in team-based learning in person, but for the Class of 2022 it was held virtually. This change may have contributed to their lower confidence levels compared to previous classes. More efforts should be made to provide students with in-person opportunities to demonstrate their abilities for interprofessional practice in order for students to be “team ready” prior to APPEs.

IPPEs were greatly affected by the pandemic, which resulted in a large number of rotations being canceled. To meet the shortage of available sites and preceptors, many faculty were asked to precept students for virtual rotations plus a newly hired clinical staff pharmacist also developed a remote and hybrid institutional IPPE experience. Approximately 10% (37/381) of the IPPEs offered in the Class of 2022 were virtual, which meant these students had less direct interaction with patients and other health care providers during these experiences. Communication with patients and preceptors were performed via phone or video conferencing and students completed their patient care services remotely. Practicing these activities is known to increase student independence [21]. On the other hand, the students from the Class of 2021 and 2020 completed their IPPEs before the pandemic shutdowns occurred, and therefore were not impacted by the IPPE shortages. 

The Office of Professional Experience Programs conducts a two-hour APPE orientation session for the P3 students shortly before the end of the Spring semester and the start of the first APPE block. This orientation is typically conducted in person and students have opportunities to ask questions and receive guidance from faculty. For the Class of 2022, the orientation information was pre-recorded and one hour in length. Students were instructed to watch the recording and invited to virtual office hours to ask questions; however, this session was not well-attended. The P3 class board also organized a virtual presentation with a panel of APPE students (P4 students in 2021) to share their APPE experiences and provide the P3 students with advice for APPE preparation and pearls for APPE success. This session was also not well-attended by the P3 students.

The Class of 2022 performed well overall in the program; they still reported less confidence than their peers in other cohorts. The Class of 2022 submitted their responses to the APPE Readiness survey in the midst of the pandemic after completing a full academic year virtually, while the Class of 2021 completed the same survey at the very beginning of the pandemic. The perceived low confidence in APPE readiness was a short-lived outcome that seemed to change once they started APPEs, as indicated on their midpoint self-evaluations during block 1 APPE.

The effect of the increase in virtual IPPEs for the Class of 2022 is difficult to quantify. Even though it appears that only a small percentage of IPPEs were virtual, many other IPPEs may have had to alter their patient care and interprofessional activities of which the Office of Professional Experiential Programs is not aware. Limitations on a students’ opportunities to practice communicating with patients, other healthcare professionals, and their preceptors may have been a factor that affected their confidence levels for APPE readiness, especially since more advanced IPE activities such as active engagement in teamwork collaboration are expected during APPEs [22]. 

Survey responses indicated that students in the Classes of 2020, 2021 and 2022 shared similar primary work setting experiences and thus it does not appear that student employment in any particular work setting had a negative effect on the class of 2022′s APPE readiness confidence levels. Overall, the students in the Class of 2022 reported working fewer weekly hours throughout their P1–P3 years. Having worked fewer hours may translate to less work experience which can lead to lower confidence in APPE preparedness. As reported elsewhere [23], some students in the Class of 2022 had to work extra hours during the pandemic being the primary force behind COVID-19 testing and vaccine administration at their practice sites, while others may have worked less due to social distancing protocols in various institutions. Their roles at work may have also been altered as many pharmacy personnel were required to work remotely and patient care services limited in-person interactions. Isolation from supervisors, team members and patients may have contributed to students feeling unprepared to start APPEs. Even though the students in the three cohorts worked in similar practice settings, the students in the Class of 2022 likely experienced more stressful work conditions working during a pandemic under heightened safety procedures, having to constantly adapt to constantly changing COVID-19 protocols, being primary COVID-19 vaccine providers, while carrying personal, family, and academic responsibilities. 

The response rates for the APPE Readiness Survey for the Classes of 2020, 2021 and 2022 were low, 27.96%, 50.00%, and 30.15%, respectively. The data from the survey only represented a small proportion of each class and may not represent the majority. Efforts will be made in the future to improve the response rate for this survey.

Given the potential variability of individual student experiences throughout the pre-APPE curriculum, adopting Core EPAs across IPPEs, and aligning them with APPEs may help facilitate assessment of APPE readiness [5]. Measuring student competencies during IPPEs, simulated activities, or objective structured clinical examinations (OSCEs) would provide students with better insight on their performance abilities, which may improve their confidence prior to starting APPEs. 

Additionally, more efforts should be made by the school to support student well-being. Stress during times of uncertainty throughout the pandemic may have affected the student’s abilities to adapt and learn, therefore impacting their confidence to perform [24].

## 5. Conclusions

Our response to the low confidence in APPE readiness led us to find that what and how we taught the students was sufficient and appropriate; however, the general combined experience of virtual education, remote and unproctored summative assessments, virtual IPPEs and reduced employment hours and professional interactions affect students’ self-perceived APPE readiness. Moving forward into the post-COVID era of teaching and learning, we must acknowledge that regardless of the explicit benefits of moving knowledge-level material online, expecting students to complete learning at their own pace and writing assessment questions that require critical thinking and are conducive to an open-book exam, students will perceive their experience as “less than” unless there are frequent in-person and on-site skills assessments that help improve their confidence. The challenge here is to ensure students’ confidence in the curriculum, their abilities, and conveying that quality of education is not compromised when teaching and learning is changed in a thoughtful way.

## Figures and Tables

**Table 1:** P3 students’ reported confidence in ability-based outcomes (ABOs).

Ability-Based Outcomes (ABOs) (*n* = 60)	Not Confident	Unsure	Confident & Very Confident
Promote Health & Wellness	0 (0.00%)	1 (1.67%)	59 (98.33%)
Self-awareness (*n* = 59)	0 (0.00%)	1 (1.69%)	58 (98.31%)
Professionalism in patient interations (*n* = 59)	0 (0.00%)	2 (3.39%)	57 (96.61%)
Professionalism in interactions with society	0 (0.00%)	3 (5.00%)	57 (95.00%)
Demonstrate accountability and ownership in all professional activities	0 (0.00%)	4 (6.67%)	56 (93.33%)
Professionalism in healthcare provider interactions	0 (0.00%)	4 (6.67%)	56 (93.33%)
Cultural Sensitivity	0 (0.00%)	4 (6.67%)	56 (93.33%)
Exhibit behaviors & values that are consistent with the trust given to the profession	0 (0.00%)	4 (6.67%)	56 (93.33%)
Communication	0 (0.00%)	5 (8.33%)	55 (91.67%)
Patient Advocacy	0 (0.00%)	8 (13.33%)	52 (86.67%)
Develop, Integrate, and Apply Knowledge from the Foundational Sciences	1 (1.67%)	7 (11.67%)	52 (86.67%)
Provide Patient Centered Care	1 (1.67%)	9 (15.00%)	50 (83.33%)
Problem Solving	1 (1.67%)	10 (16.67%)	49 (81.67%)
Evaluate the Scientific Literature	0 (0.00%)	11 (18.33%)	49 (81.67%)
Explain Drug Action	0 (0.00%)	12 (20.00%)	48 (80.00%)
Leadership	1 (1.67%)	11 (18.33%)	48 (80.00%)
Educator	0 (0.00%)	13 (21.67%)	47 (78.33%)
Interprofessional Collaboration	2 (3.33%)	11 (18.33%)	47 (78.33%)
Provide Population-Based Care	1 (1.67%)	13 (21.67%)	46 (76.67%)
Optimize Safety & Efficacy of Medication Use Systems	1 (1.67%)	15 (25.00%)	44 (73.33%)
Advance Population Health & Patient Centered Care	1 (1.67%)	16 (26.67%)	43 (71.67%)
Solve Therapeutic Problems (*n* = 59)	2 (3.39%)	15 (25.42%)	42 (71.19%)
Technological Resources (*n* = 59)	1 (1.69%)	20 (33.90%)	38 (64.41%)
Physical Resources (*n* = 59)	1 (1.69%)	21 (35.59%)	37 (62.71%)
Innovation & Entrepreneurship	3 (5.00%)	20 (33.33%)	37 (61.67%)
Human Resources (*n* = 59)	3 (5.08%)	23 (38.98%)	33 (55.93%)
Financial Resources	5 (8.33%)	28 (46.67%)	27 (45.00%)

**Table 2:** P3 Students’ confidence in ACPE Standards Appendix 1 Topics.

Appendix 1 Topics	Confident or Very Confident		
	Class of 2022	Class of ’20 & ’21 Combined	Percent Δ
Biochemistry	66.67%	88.03%	−21.36%
Pharmacy Law & Regulatory Affairs	60.00%	71.79%	−11.79%
Immunology	65.00%	76.22%	−11.22%
Human Physiology	71.67%	78.17%	−6.50%
Pharmacogenomics/genetics	56.67%	61.97%	−5.31%
Public Health	77.97%	80.99%	−3.02%
Medical Microbiology	63.33%	65.28%	−1.94%
Medication Dispensing, Distribution, & Administration	88.14%	88.67%	−0.53%
Professional Communication	91.67%	92.20%	−0.53%
Practice Management	56.67%	57.04%	−0.38%
Ethics	95.00%	92.86%	2.14%
Pharmacoepidemiology	60.00%	57.86%	2.14%
Biostatistics/research design	54.17%	50.21%	3.95%
Patient Assessment	83.05%	77.78%	5.27%
Natural Products and Alternative and Complementary Therapies	57.63%	52.11%	5.51%
Extemporaneous Compounding	65.00%	57.64%	7.36%
Pharmacoeconomics	61.67%	52.82%	8.85%
Medicinal Chemistry	71.67%	60.00%	11.67%
Pharmaceutics/Biopharmaceutics	70.00%	55.94%	14.06%
Pharmacotherapy	84.75%	69.72%	15.03%
Pharmacology and Toxicology	78.33%	62.89%	15.45%
Healthcare Systems	71.67%	52.45%	19.22%
Pharmacokinetics/Clinical Pharmacokinetics	76.47%	56.16%	20.31%

**Table 3:** P3 students’ confidence in the didactic curriculum.

Class of 2022 vs. (2021 & 2020) Ranked by Strongly Agree/Agree (2022 < 2021&2020)			
Topic	Agree/ Strongly Agree		
	Class of 2022	Class of 2021 & 2020	Percent Δ 2022 vs. (2021 & 2020)
Assess the health needs of a given patient population.	44/59 (74.58%)	114/140 (81.43%)	**−6.85%**
Provide patient-centered care based on evidence-based best practices; design strategies and solutions to address patient-care problems.	140/176 (79.55%)	273/321 (85.05%)	**−5.50%**
Apply knowledge from the foundational clinical, pharmaceutical, and biomedical sciences to the provision of patient care.	92/118 (77.97%)	192/236 (81.36%)	**−3.39%**
Effectively communicate (verbal, non-verbal, written) when interacting with individuals, groups, and organizations.	49/59 (83.05%)	163/191 (85.34%)	**−2.29%**
Act in a manner consistent with the trust given to pharmacists by patients, other healthcare providers, and society.	52/59 (88.14%)	127/142 (89.44%)	**−1.30%**
**Class of 2022 vs (2021 & 2020) Ranked by Strongly disagree/disagree (2022 > 2021&2020)**			
**Topic**	**Disagree/ Strongly Disagree**		
	**Class of 2022**	**Class of 2021 & 2020**	**Percent Δ** **2022 vs. (2021 & 2020)**
Provide patient-centered care based on evidence-based best practices; design strategies and solutions to address patient-care problems.	10/176 (5.68%)	9/321 (2.80%)	**2.88%**

**Table 4:** Average preceptor final evaluation scores on APPE student performance per ABO.

Ability Based Outcomes (ABOs)	Average Final Evaluation Scores
Provided patient centered care	4.42 (*n* = 3560)
Promoted health and wellness	4.39 (*n* = 3641)
Provided population-based care	4.31 (*n*= 3407)
Develop, integrate, and apply knowledge from the foundational sciences	4.28 (*n* = 3955)
Evaluate the scientific literature	4.29 (*n* = 3949)
Explain drug action	4.21 (*n* = 3779)
Solve therapeutic problems	4.17 (*n* = 3661)
Advance population health and patient centered care	4.25 (*n* = 3501)
Demonstrate problem solving skills	4.34 (*n* = 4020)
Demonstrate educator skills	4.34 (*n* = 3868)
Engage in patient advocacy	4.40 (*n* = 3585)
Demonstrate inter-professional collaboration	4.60 (*n* = 4173)
Demonstrate cultural sensitivity	4.54 (*n* = 4027)
Demonstrate effective communication skills	4.47 (*n* = 4143)
Exhibit behaviors and values that are consistent with the profession of pharmacy	4.67 (*n* = 4320)
Demonstrate professionalism in interactions with patients	4.68 (*n* = 3574)
Demonstrate professionalism in interactions with other healthcare providers	4.66 (*n* = 4231)
Demonstrate professionalism in interactions with society	4.64 (*n* = 3809)
Exhibit behaviors that demonstrate accountability and ownership in all professional activities	4.59 (*n* = 4271)
Optimize safety and efficacy of medication use systems	4.36 (*n* = 3161)
Manage human resources	4.36 (*n* = 1717)
Manage financial resources	4.31 (*n* = 1447)
Manage technological resources	4.49 (*n* = 3372)
Manage physical resources	4.44 (*n* = 2675)
Demonstrate self-awareness	4.53 (*n* = 4207)
Demonstrate leadership	4.26 (*n* = 3729)
Innovation and entrepreneurship	4.25 (*n* = 3128)

## Data Availability

The data presented in this study are available on request from the corresponding author. The data on aggregate academic performance are not publicly available.
